# Aluminum–Titanium Bilayer for Near-Infrared Transition Edge Sensors

**DOI:** 10.3390/s16070953

**Published:** 2016-06-23

**Authors:** Lapo Lolli, Emanuele Taralli, Chiara Portesi, Mauro Rajteri, Eugenio Monticone

**Affiliations:** Istituto Nazionale di Ricerca Metrologica, St. delle Cacce 91, Torino 10135, Italy; e.taralli@inrim.it (E.T.); c.portesi@inrim.it (C.P.); m.rajteri@inrim.it (M.R.); e.monticone@inrim.it (E.M.)

**Keywords:** photon counting detector, superconductivity, optics

## Abstract

Transition-edge sensors (TESs) are single photon detectors attractive for applications in quantum optics and quantum information experiments owing to their photon number resolving capability. Nowadays, high-energy resolution TESs for telecommunication are based on either W or Au/Ti films, demonstrating slow recovery time constants. We report our progress on the development of an Al/Ti TES. Since bulk aluminum has a critical temperature (*T*_c_) of ca. 1.2 K and a sufficiently low specific heat (less than 10^−4^ J/cm^3^K^2^), it can be employed to produce the sensitive material for optical TESs. Furthermore, exploiting its high *T*_c_, Al-based TESs can be trimmed in a wider temperature range with respect to Ti or W. A first Al/Ti TES with a *T*_c_ ≈ 142 mK, investigated from a thermal and optical point of view, has shown a response time constant of about 2 μs and single photon discrimination with 0.34 eV energy resolution at telecom wavelength, demonstrating that Al/Ti films are suitable to produce TESs for visible and NIR photon counting.

## 1. Introduction

A transition-edge sensor is a superconducting phase thermometer, able to detect the number of incident photons through its intrinsic energy resolution capability. The core of a TES is the superconducting thin film: it is biased in its transition region, and, in this way, the film works as a sensitive thermometer measuring the temperature change due to the photon absorption.

Presently, TESs are employed as microcalorimeters and bolometers to detect radiation in a wide spectral region, from millimeter waves to gamma rays. So far, TESs for IR-visible range have been fabricated using different superconducting materials: single layer-based TESs use tungsten [1], hafnium [2], or titanium [3], whereas bilayer-based TESs use titanium proximized by gold [4], palladium [5], or molybdenum proximized by gold [6].

In superconducting and normal metal bilayer thin films, the critical temperature can be theoretically trimmed from 0 to the *T*_c_ of the bulk superconductor by changing the layer thickness ratio [7]. In this way, it is possible to control the most important characteristics of a TES detector [8] that depend on temperature: heat capacity, response time constant, and energy resolution.

The aluminum critical temperature is the highest among tungsten, hafnium, molybdenum, and titanium, so its use as superconducting material, with titanium as a normal material, allows for the tuning of the *T*_c_ in a wide temperature range, from a few tens of mK up to 1.2 K, selecting the appropriate values of layer thicknesses.

TESs based on an Al/Ti bilayer have already been used as bolometers for astrophysical applications at far-infrared and millimeter wavelengths [9,10]. Recently, also in the optical range, a characterization of a suspended TES bolometer has been published [11]. However, in this case, the high level of noise did not allow for the discrimination of single photons at telecommunication wavelengths.

The microcalorimeter prototype here presented has been developed to demonstrate the photon-number resolving (PNR) capability of TES devices based on Al proximized by Ti, in the optical and NIR spectral range. This work reports thermal and optical properties, compared with simulation data, of an Al/Ti TES working above 140 mK, with an active area of 100 µm^2^ and Nb wirings. With this low *T*_c_ and wide active area, this prototype has demonstrated to be a photon number resolved up to eight photon states at 1545 nm. Al/Ti TESs could be utilized to cover specific needs, such as lower response time constants and higher saturation energy.

## 2. Materials and Methods

The characteristics of superconducting thin films, which serve as sensitive material, are very important for TES microcalorimeters. The main parameters are the critical temperature *T*_c_ and the transition width *ΔT*_c_ between the superconducting and the normal phase. Since *T*_c_ and *ΔT*_c_ depend both on the quality of each layer and the interface transparency between the layers, an excellent control of the deposition process is essential to fabricate TESs with good performances. Therefore, the bilayer films of aluminum and titanium have been produced with different thicknesses of Ti and Al, following procedures similar to one described in [12]: high vacuum deposition with an e-gun (base pressure < 10^−5^ Pa) and a lift-off of Al/Ti to define active areas ranging between 100 μm^2^ and 400 μm^2^.

**Figure 1:**
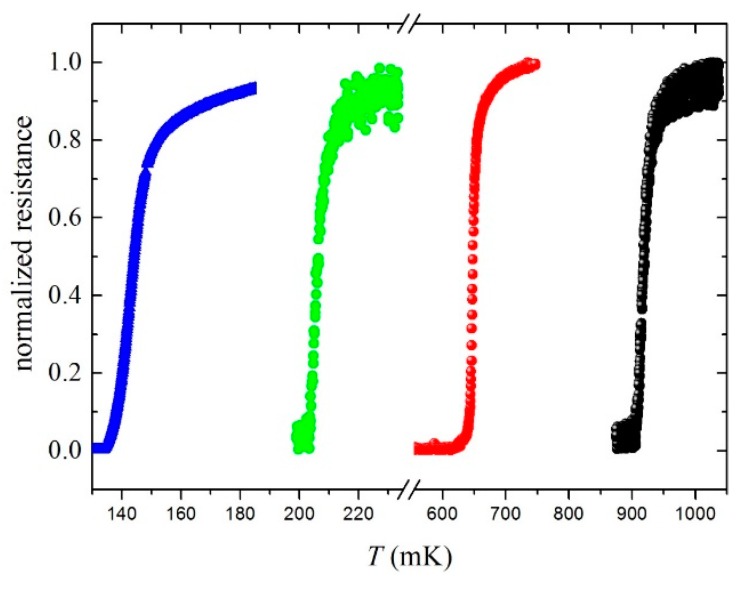
Critical temperatures of different Al/Ti transition-edge sensors (TESs) ranging between 0.14 K and 1 K.

Superconducting wirings are defined by an RF sputtering deposition of 40 nm of Nb, followed by optical lithography. Before the Nb deposition, the Ti film surface was sputter-cleaned to reduce the contact resistance between Nb and Ti. Wirings with a higher *T*_c_ limit the outdiffusion of hot electrons from Al/Ti into electrodes because of Andreev reflection [13]. Figure 1 shows the critical temperatures of four TES prototypes with areas of 10 µm × 10 µm, measured by the 4-wires technique. The samples have an Al layer of 40 nm, while the Ti thickness ranges from 5 to 15 nm.

## 3. Results

**Figure 2:**
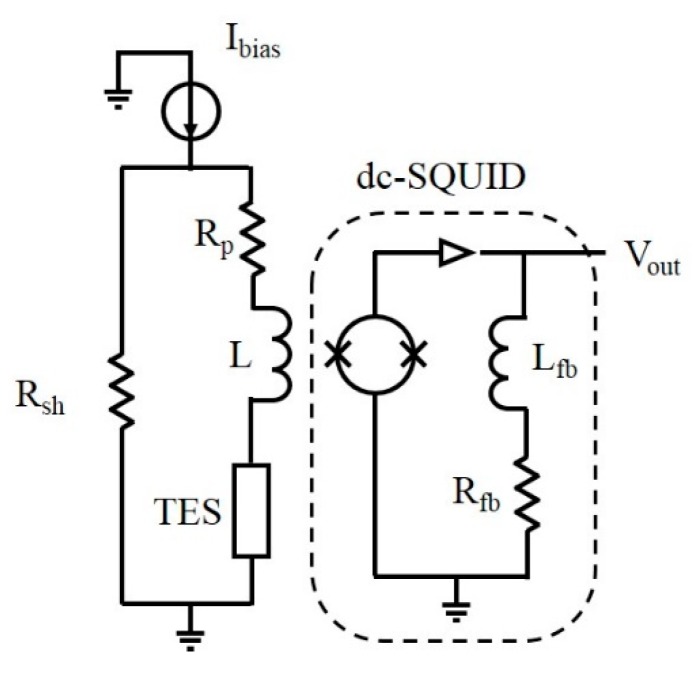
Scheme of the electronic bias circuit with the TES, the shunt resistance (*R*_sh_), the parasitic resistance (*R*_p_), and the read-out line exploiting an array of dc-SQUID as current amplifier.

The device with a critical temperature of 0.142 K (in Figure 1) has an active area of 10 µm × 10 µm and a thickness of 55 nm (15 nm of Ti and 40 nm of Al). The device was put in a standard voltage‑biased circuit and linked to a dc-SQUID to read out the device current [14] (Figure 2). The circuit was cooled down through a dilution refrigerator with a base temperature of 35 mK.

**Figure 3:**
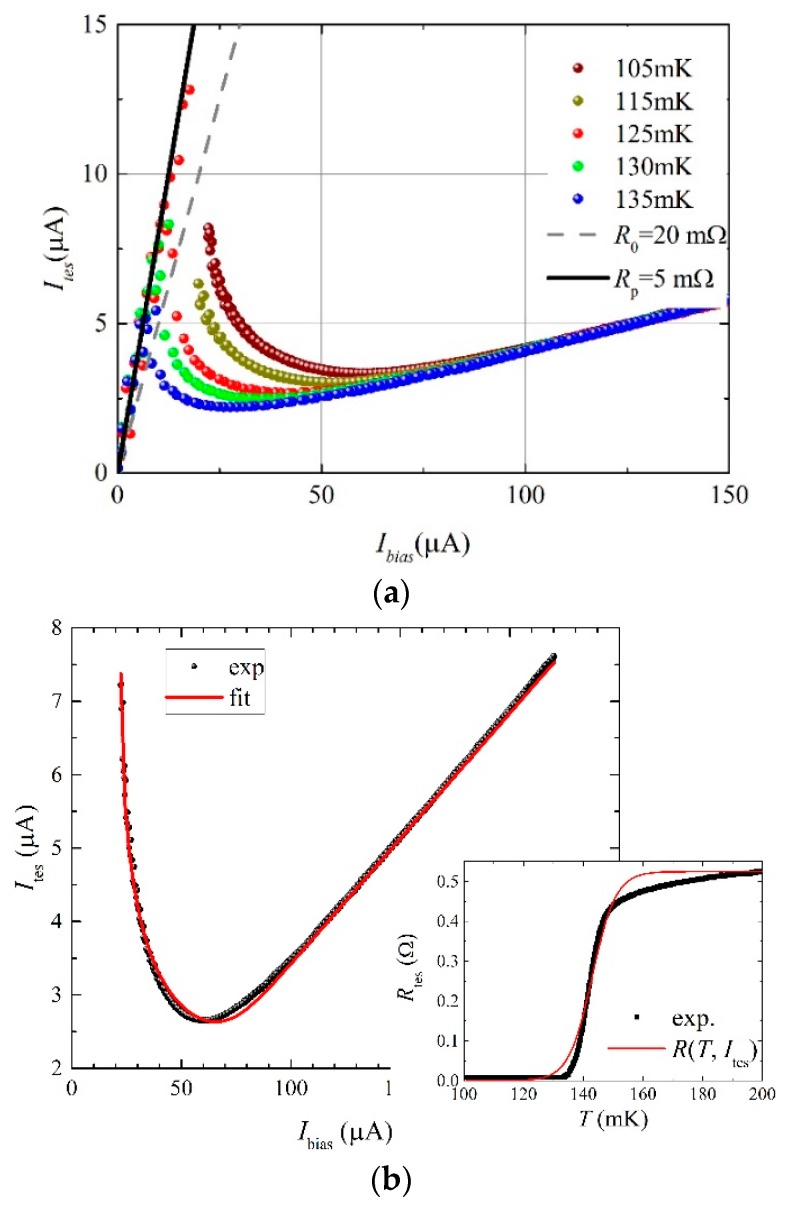
TES bias curve at different bath temperatures. (**a**) Dots are experimental data of *I*_tes_ vs. *I*_bias_; lines are the values at the working point *R*_0_ and parasitic resistance *R*_p_. (**b**) *I*_tes_ vs. *I*_bias_ experimental data and fit at 108 mK; the inset shows a comparison between the *R*(*T*,$\;I_{\text{tes}}$) curve calculated by Equation (2) and the corresponding one of Figure 1.

Figure 3a shows the experimental values of the currents through the device (*I*_tes_) as a function of the bias currents (*I*_bias_), for the bath temperature range 105 mK ≤ *T*_b_ ≤ 135 mK (dots). The detector working point is typically tuned along these curves with the aim to optimize some detector features for specific experimental applications. In the case of this work, the aim is to demonstrate the PNR capability of the Al/Ti TES, so the working point has been experimentally selected to obtain the best energy resolution.

For each curve of Figure 3a (*I*_tes_ vs. *I*_bias_), the dissipated power (*P*) is calculated at the corresponding resistance of the working point *R*_0_ = 20 mΩ (4% of its normal resistance, *R*_n_ ≈ 0.5 Ω). By fitting the experimental data, with the relation *P* = *k*(*T*_c_ *^n^* − *T*_b_ *^n^*), it is found that *n* ≈ 5, as expected for electron-phonon conduction in metals with small volume and high power densities [13], and from *k* the thermal conductance *G* = *nkT*_c_ *^n^*^−1^, is calculated approximately 0.1 nW/K.

The fits of the bias curve (continuous line in Figure 3b) is obtained imposing the equilibrium state at the heat balance equations [4]:

(1) $$\{\begin{array}{c} \left(I_{\text{bias}}\;–\;I_{\text{tes}}\right)R_{\text{sh}}\;-\;I_{\text{tes}}\left(R_{p}\;+\;R\left(T,I_{\text{tes}}\right)\right)=\;0\\ R\left(T,\;I_{\text{tes}}\right)I_{\text{tes}}^{2}\;-\;k\left(T^{n}-\;T_{b}^{n}\right)=\;0 \end{array}$$

where *R*_sh_ is the shunt resistance, *R*_p_ is the parasitic resistance, and using a hyperbolic tangent function of current and temperature to represent *R*(*T*, $I_{\text{tes}}$) [4]:

(2) $$R\left(T,\;I_{\text{tes}}\right)=\frac{R_{n}}{2}\left[1+\tanh\left(\frac{T-T_{c}+\xi \;I_{\text{tes}}}{D}\right)\right]$$

where *ξ* takes into account the current dependence of the resistance, and *D* models the transition width. The fit procedure is obtained minimizing the chi-square test between the expected and simulated *I*_tes_ vs. *I*_bias_ curves.

At *T*_b_ = 108 mK, with *n* = 5 and with *R*_sh_ = 22 mΩ, (Figure 3b) the best fit produces, for the free parameters, the following results: *R*_p_ = 5 mΩ; *ξ* = 2.5 K/A; *D* = 7.9 mK; and, finally, *k* = 47 nW/K^5^. This latter value is in agreement with results from the dissipated power method and confirms the goodness of fit procedure. The inset of Figure 3b compares the *R*(*T*,$\;I_{\text{tes}}$) behavior obtained from the four-wire technique measurement with Equation (2). The mismatch at the top of the transition is due to the proximity effect, which is not taken into account in the model of Equation (2).

**Figure 4:**
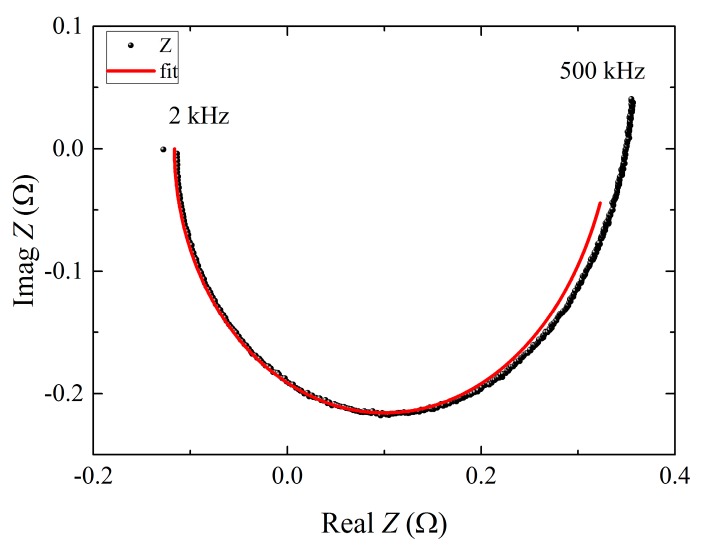
Measured (dots) device impedance *Z* at *R*_0_ bias point and the corresponding fit (line) between 2 kHz and 500 kHz of frequencies.

Figure 4 (dots) shows device impedance (*Z*) measurements [15] as a function of frequency performed at the same working point of *I_0_* = 19 µA, which corresponds to bias the device at *R*_0_ = 4% *R*_n_.

In the frequency domain of 2 kHz ÷ 500 kHz, the fit of the experimental data (red line in Figure 4) [15] provides the following devices parameters that cannot be directly measured. The fit procedure has been applied simultaneously on the frequency behavior of the real and imaginary part of the impedance measurement, minimizing the chi-square test.

The logarithmic derivative of the TES resistance with respect to temperature at constant current *α* = (*T*_0_/*R*_0_)(∂*R*/∂*T*)|*_I_* = 10, the current sensitivity *β* = (*I*_0_/*R*_0_)(∂*R*/∂*I*)|*_T_* = 15, and the electronic heat capacity *C*_e_ = 0.32 fJ/K. This latter value is compatible with the value estimated by considering literature data [16] and the volume of deposited Al and Ti, ca. 0.72 fJ/K.

Two of the main characteristics of single photon detectors are the energy resolution, i.e., how well it discriminates a photon, and the response time constant. The device intrinsic energy resolution is [17]

(3) $$\Delta E_{FWHM}=2\sqrt{2\ln(2)}\cdot \sqrt{4k_{b}{T_{c}}^{2}\frac{C_{e}}{\alpha }\sqrt{\left(1+r\right)\frac{n}{\varphi }\left(\frac{1}{2}\left(1+\frac{{T_{b}}^{2}}{{T_{c}}^{2}}\right)+\frac{n}{\alpha^{2}\varphi }\left(1+ra^{2}\right)\right)}}$$

where *φ* = 1 − (*T*_b_/*T*_c_)*^n^*, *r* = *R*_sh_*T*_b_/(*R*_0_*T*_c_), and *a* = (1 + *α*ϕ/*n*). Meanwhile, the theoretical response time constant is [17]

(4) $$\tau =\frac{C_{e}}{G}{\left\{1+\frac{\alpha }{n}\left[1-{\left(\frac{T_{b}}{T_{c}}\right)}^{n}\right]\right\}}^{-1}$$

By substituting in Equations (3) and (4), the values obtained by the fits of the bias curve and impedance measurement, *ΔE*_FWHM_, results in 0.17 eV and *τ* = 1.3 μs.

**Figure 5:**
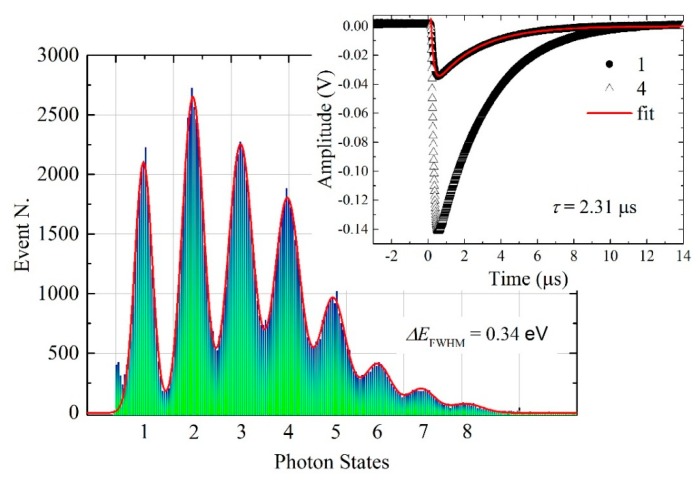
Eight photon states are distinguishable at 1545 nm with an energy resolution of 0.34 eV. The inset shows the single and the four photons averaged pulses; by the pulse fit the response time constant is 2.31 µs.

The aim of this work is to demonstrate the PNR capacity of an Al/Ti TES, so this device was stabilized in a thermal bath of 108 mK and tested for single photon detection at telecommunication wavelengths. To irradiate the TES, a single-mode optical fiber was aligned at room temperature over the active area [14]. The photon source was a 5 kHz repetition rate pulsed laser, with a pulse width of 35 ps, at 1545 nm. Figure 5 shows the histogram of the photon discrimination: it is possible to distinguish up to eight Gaussian peaks representing the corresponding photon states.

By the histogram of Figure 5, the energy resolution has been calculated considering the full width at half maximum of the first photon state peak [4]: *ΔE*_FWHM_ = 0.34 eV. The worsening with respect to theoretical value is probably due to a lack of full overlap of the optical fiber tip onto the device active area, which causes photons to be deposited on the edge of the detector, absorbed by Nb wirings or by the substrate near the TES [4].

The inset of Figure 5 shows the single photon averaged pulse (dots). The fit obtained by a double exponential equation [18] provides the response time constant of *τ* = 2.31 µs, close to the value obtained by the theoretical estimation. The areas under the averaged photon pulses represent the collected energy, *E*_col_. These values are compared with the incident photon energy (0.8 eV), finding an overall collection efficiency of ε ≈ 0.4. This number is far from those of Ti/Au TES, ε ≈ 0.9 [19], and Ti/Pd TES, ε ≈ 0.7 [20], but close to that reported for W TES [21]. The different thicknesses of TESs and the diffusivity of Al compared with Au and Pd could explain the energy loss outside the active areas.

## 4. Conclusions

An Al/Ti bilayer, compared with that of Ti/Au and Ti/Pd, is a valid alternative solution for producing TESs able to detect telecom photons down to the single photon regime, with potential applications beyond astrophysics at far-infrared and millimeter wavelengths.

Due to the low *T*_c_ with respect to pure aluminum, the Al/Ti TES presented here has a response time constant of the order of microseconds, with an energy resolution of 0.34 eV, and discriminates up to eight photon states. Al/Ti bilayers are promising because, by reducing the device active area at 1 μm^2^ and raising the working temperature up to 700 mK, the response time constant should reduce to only hundreds of nanoseconds without worsening the energy resolution. This possibility to trim the *T*_c_ over a wide temperature range could be very useful for tailoring TES detectors for specific detection experiments.
